# Merging familiar and new senses to perceive and act in space

**DOI:** 10.1007/s10339-021-01052-3

**Published:** 2021-08-19

**Authors:** Marko Nardini

**Affiliations:** Department of Psychology, Durham University, Science Site, Durham, DH1 3LE UK

**Keywords:** Spatial cognition, Multisensory development, Development, Sensory augmentation, Bayesian

## Abstract

Our experience of the world seems to unfold seamlessly in a unitary 3D space. For this to be possible, the brain has to merge many disparate cognitive representations and sensory inputs. How does it do so? I discuss work on two key combination problems: coordinating multiple frames of reference (e.g. egocentric and allocentric), and coordinating multiple sensory signals (e.g. visual and proprioceptive). I focus on two populations whose spatial processing we can observe at a crucial stage of being configured and optimised: children, whose spatial abilities are still developing significantly, and naïve adults learning new spatial skills, such as sensing distance using auditory cues. The work uses a model-based approach to compare participants’ behaviour with the predictions of alternative information processing models. This lets us see when and how—during development, and with experience—the perceptual-cognitive computations underpinning our experiences in space change. I discuss progress on understanding the limits of effective spatial computation for perception and action, and how lessons from the developing spatial cognitive system can inform approaches to augmenting human abilities with new sensory signals provided by technology.

## Introduction

Our experience of the world seems to unfold seamlessly in a unitary 3D space. For this to be possible, the brain has to merge many disparate cognitive representations and sensory inputs. How does it do so? Here, I review work on two key combination problems: coordinating multiple frames of reference (e.g. egocentric and allocentric) and combining multiple sensory signals (e.g. visual and proprioceptive). I focus on two populations whose spatial processing we can observe at a crucial stage of being configured and optimised: children, whose spatial abilities are still developing significantly, and naïve adults learning new spatial skills, such as sensing distance using novel auditory cues.

## Coordinating spatial frames of reference

### Spatial frames of reference

Spatial relationships can be stored in different frames of reference, with advantages for specific tasks. To open my car door, it is most useful to store where it is relative to my hand (a body- or self- referenced, *egocentric* representation). In contrast, to find the car in the car park, perhaps from a new viewpoint, it is most useful to store where it is relative to stable external landmarks (an externally referenced, *allocentric* representation). The brain represents spatial representations with different coordinate frames using different specialised substrates (review, Burgess 2008)—for example, those in body-referenced frames useful for guiding immediate action in parietal cortex (Bremmer et al. 1997), and those in frames using external landmarks in the hippocampus (Hartley et al. 2014).

### Development of spatial frames of reference

Since Piaget’s pioneering investigations of spatial cognitive development (Piaget and Inhelder 1956), it has been evident that children achieve competence at egocentric responses and tasks earlier than allocentric ones. Particularly, when egocentric and allocentric responses conflict, young children tend to follow an incorrect egocentric strategy. For example, in studies by Acredolo (Acredolo 1978; Acredolo and Evans 1980), younger infants who learned to turn to one side (e.g. *their* right) to find a target, and were then moved and rotated 180°, persevered with this now incorrect egocentric response. This points to the multiple challenges of encoding more complex allocentric versus simpler egocentric spatial relationships, updating representations correctly to account for own movement, and selecting the correct reference frame when different frames conflict (more discussion: Nardini et al. 2009, and below).

### Development: coordinating multiple reference frames

Most of the time, multiple potential encodings or frames—which may be more or less useful for a specific task—are available. Beginning in 2006, our studies addressed the question when and how multiple reference frames are coordinated in development. In an initial study, 3–6-year olds attempted to recall the locations of objects on an approximately 1m^2^ board incorporating small surrounding landmarks (Nardini et al. 2006). Board and/or participant were moved between hiding and recall in a factorial design that varied the validity of (1) the self, (2) the wider room, and (3) the small surrounding landmarks as a basis for recall. Children were already competent from age 3 years when self- and/or room-based reference frames were available, but only above chance from 5 years at using the surrounding landmarks alone (and disregarding the other frames). Subsequent modelling of responses indicates that at intermediate ages, children’s responses are a mixture between using the incorrect frames and the correct one (Negen and Nardini 2015). A highly controlled version of the same task using VR—in which children no longer interact with a miniature moving array, but are immersed in the virtual test environment (Negen et al. 2018a) reached the same conclusion. Simple (e.g. body-referenced) representations are reliably used from a young age, but when these are not valid, correctly coordinating and using only relevant landmarks to respond emerges later, at 4–5 years of age.

### Development: coordinating multiple landmarks

Tracing the earliest ages at which allocentric recall (i.e. using only external landmarks) is demonstrably above chance identifies a starting point for allocentric abilities, but these very earliest abilities may be based only on very simple or partial information about external landmarks. For example, in Negen et al. (2018a), the earliest above-chance use of the allocentric frame could be explained by encoding position just along one axis of the space—far short of a fully accurate spatial representation. Similarly, allocentric recall that can be based on roughly matching visual features emerges earlier than that requiring strict representation of spatial relationships (Nardini et al. 2009). A VR study of 3-to-8-year olds’ recall with respect to several distinct landmarks asked how abilities to coordinate these develop (Negen et al. 2019a). The study looked for markers of performance beyond that explicable by use of just the single nearest landmark. The results showed that until around 6 years, allocentric performance was supported by use of a single landmark—a strategy better than egocentric, but still subject to significant errors (e.g. mirror reversals). Only after 6 years was there evidence for coordination of multiple landmarks to improve precision and avoid such errors. Interestingly, however, this was also moderated by the complexity of the environment—in an extremely simple (less naturalistic) space, there was earlier evidence for coordination of multiple landmarks.

### Coordinating multiple reference frames and landmarks: developmental mechanisms and bottlenecks

These studies reveal crucial computational changes in spatial recall during early life. We see a progression from reliance on simple (body-based/egocentric) encodings, to those using simple elements of the external environment (e.g. single landmarks, or features of landmarks), to those coordinating multiple landmarks. The competence of typical adults at perceiving and acting flexibly in space emerges from this long developmental trajectory. On comparable experimental tasks, clinical groups with spatial difficulties (e.g. Williams Syndrome) appear to remain at levels of development typical of pre-allocentric children (e.g. Nardini et al. 2008a), as do adult hippocampal patients (King et al. 2002). What are the developmental mechanisms, and what bottlenecks hold back younger children (or clinical groups) from flexible spatial recall? The degree to which these changes represent either reshaping of abilities to *encode and represent* the relevant information (e.g. by the hippocampus), or abilities to *correctly select the relevant encoding* (disregarding irrelevant cues or reference frames) is one key question for future research. Initial evidence that individual differences linked to inhibitory control are one predictor of performance (Negen et al. 2019a) suggests that not only encoding, but also selection plays a role. Evidence in the same study that a simpler environment shows earlier development also suggests a role for processes of attention and cue selection. These findings raise interesting questions about how closely the present coordination problems in spatial cognitive development are linked to development of more general, central, cognitive capacities, such as inhibition or cognitive control.

## Coordinating multiple sensory signals

### Multisensory processing of spatial information

We sense the world using multiple channels of sensory input, including visual, auditory, and haptic. The challenge of situating ourselves in space includes coordinating and combining these disparate information sources. For example, for dealing with changes of viewpoint (see above), visual information is useful for detecting the new viewpoint (e.g. using visual landmarks) and potentially for tracking own movement between the different viewpoints (e.g. using optic flow). Non-visual (e.g. vestibular and kinesthetic) information also crucially helps track own movement to account for viewpoint changes (Simons and Wang 1998; Wang and Simons 1999), including during development (Nardini et al. 2006; Negen et al. 2018a). This is evident in the studies just mentioned because when viewpoint changes happen in absence of movement-related information (e.g. a new viewpoint is presented, but the participant did not walk there), accuracy is poorer in adults and takes longer to be above chance in childhood.

### Measuring combination of multisensory spatial signals

The evidence reviewed above for the role of movement, as well as vision, comes from spatial tasks that create large cue conflicts. In key test conditions, a viewpoint change is experienced without the corresponding movement—i.e. the environment is rotated in front of the participant, or the participant is virtually ‘teleported’. This leaves unclear the extent to which performance is poor because of (a) the absence of useful movement information, or (b) an incorrect reliance on the (erroneous) movement information that states that no viewpoint change has occurred. We saw that young children just mastering these tasks switch between the latter erroneous strategy and one that correctly disregards movement information (Negen and Nardini 2015), and that performance on a related task is predicted by individual differences in inhibitory control (Negen et al. 2019a). To more clearly determine how spatial signals and cues interact, a more recent approach (Cheng et al. 2007) applies Bayesian decision theory to questions about how spatial information is combined. This avoids selection and conflict problems and also lets us measure the degree to which using two signals together leads to the precision benefits expected for a rational (Bayesian) ideal decision-maker. The approach essentially (see Ernst and Banks 2002; Rohde et al. 2016) varies the availability of cue 1 and cue 2 across conditions (testing cue 1 alone, cue 2 alone, and cues 1 + 2 together) to test for Bayesian precision benefits. It also uses *small* conflicts (cue 1 vs. cue 2 indicate slightly differing target locations) to measure the relative reliance on (weighting for) each cue.

### Combination of multisensory signals for navigation

We applied this approach to a developmental navigation task (Nardini et al. 2008b). Illuminated visual landmarks in an otherwise dark room (‘cue 1’) could potentially be used together with non-visual (vestibular, kinesthetic) movement information (‘cue 2’) to return collected objects directly to their previous locations after walking two legs of a triangle (i.e. triangle completion). A Bayesian decision-maker would be measurably more precise with both cues together than with either alone. While adults met this prediction, children aged 4 and 8 years did not—they were no more precise with two cues together than with the best single cue, and the model that best explained their precision and cue weighting was one in which they selected a single cue to use on any trial, rather than combining (averaging) them. This indicates that issues with development of spatial recall in earlier tasks (e.g. Nardini et al. 2006) did not only reveal an immaturity in selecting the correct representation, but that there are also fundamental immaturities in combining multiple valid signals efficiently when these are available. The finding of efficient or near-optimal spatial cue combination in adults has been replicated and extended (Bates and Wolbers 2014; Chen et al. 2017; Sjolund et al. 2018), while the finding showing immaturity in cue combination long into childhood has been replicated in many tasks, also including more basic (e.g. table-top, non-navigational) spatial information—described next.

### Development of spatial combination of multisensory information

Basic abilities to understand multisensory correspondences and to benefit from redundant multisensory information of some kinds are present in early life (Bahrick and Lickliter 2000; Kuhl and Meltzoff 1982). However, a growing body of research shows specifically that the Bayes-like precision benefits adults experience when combining multisensory spatial signals take until around age 10 years of life or later to emerge. As well as not showing multisensory precision gains when navigating (Nardini et al. 2008b), unlike adults (Ernst and Banks 2002), children do not improve their precision at comparing the heights of bars with vision and touch together (Gori et al. 2008), in part because they overweight the less reliable cue. Similarly, unlike adults (van Beers et al. 1999), children do not improve their abilities to localise a point on a table-top with vision and proprioception together (Nardini et al. 2013). Even within the single sense of vision, unlike adults (Hillis et al. 2004), children do not combine two distinct cues to surface orientation (stereo disparity and texture) until the age of 12 years (Nardini et al. 2010); younger children’s behaviour best fits switching between following one cue or the other on any trial.

### Development of multisensory spatial combination: mechanisms and bottlenecks

These failures to achieve Bayes-like precision gains during perception long into childhood may at first seem surprising. From a decision-theoretic point of view, children—whose precision at most simple ‘unimodal’ perceptual tasks takes many years to attain adult levels—would especially stand to benefit from efficiently combining the relatively noisy information sources they have. However, to achieve efficient combination, the system must overcome a number of developmental challenges (Nardini and Dekker 2018).

#### Challenge 1: calibration

First, the different senses or signals need to be *correctly calibrated*. Initial evidence suggesting that calibration plays a role includes a study in which we found combination of visual and auditory signals to localise targets at below age 8 years in a task that improved unisensory calibration (Negen et al. 2019b).

#### Challenge 2: appropriate weighting

Second, efficient, Bayes-like combination of signals requires each to be weighted in proportion to its relative reliability, or inverse variance (Ernst and Banks 2002; Rohde et al. 2016). There is evidence for mis-weighting of signals in development, including overweighting of unreliable (Gori et al. 2008) and even completely irrelevant (Petrini et al. 2015) cues.

#### Challenge 3: neural substrates for efficient combination

A third challenge—not necessarily distinct from the above two, but expressing them at a different level of analysis, is maturation of the still poorly understood neural substrates for efficient averaging of sensory signals. It is clear that combination takes place at multiple levels of a hierarchy of sensory processing and decision-making (Rohe and Noppeney 2016), including in early ‘sensory’ areas (Gu et al. 2008). Our initial work using fMRI shows that immaturities in the earliest component of this network accompany inefficient cue combination. ‘Automatic’ combination of visual cues to 3D layout (surface slant) in early sensory (‘visual’) areas, for stimuli displayed in the background while participants carry out a different task at fixation, is present in adults (Ban et al. 2012) and in 10-to-12-year olds, but not 6-to-10-year olds (Dekker et al. 2015). Thus, acquiring efficient multisensory combination abilities for spatial judgments would seem to depend on developmental reshaping of sensory processing at a very early level.

## Enhancing human perception and action in space using new sensory signals

### Enhancing human perception and action in space: opportunities

In this final section, I sketch out applications of the work reviewed above to the newer domain of optimising human perception and action using *‘new’* sensory signals—for example, enhancing spatial abilities using new devices or sensors (Nagel et al. 2005). There is increasing evidence that the organisation of neural substrates for perception and action in space can be remarkably flexible (Amedi et al. 2017). For example, some blind individuals are expert at using click echoes to sense spatial layout, recruiting ‘visual’ cortex for perception of layout through sound (Thaler et al. 2011). Advances in wearable technology also make it increasingly feasible to provide people with novel sensors and signals. Devices to substitute or augment spatial perception via sound or vibrotactile cues have been developed and show promising signs of everyday use and reshaping perception (Maidenbaum et al. 2014). Which challenges must be met in order for approaches such as these to be integrated effectively into people’s everyday spatial cognitive repertoire?

### Enhancing human perception and action in space: challenges

There are key parallels between *children first learning to coordinate natural sensory signals* (Sect. “Coordinating multiple sensory signals”, above) and people of all ages *learning to coordinate newly learned sensory skills into their existing multisensory repertoire*. As an example, consider learning to use a new device that translates distance or depth to an auditory signal such as pitch. The three challenges identified above are also crucial here: first, achieving an *accurate calibration* of the new sense to the familiar representation of space, second, *appropriately weighting* the new signal with the old one when both provide useful information, third, at the neural level of analysis, being able to *implement* these processes in highly efficient circuits supporting subjectively effortless or ‘automatic’ perception (e.g. those in early ‘sensory’ areas).

### Enhancing human perception and action in space: initial findings

With these questions and issues in mind, we have embarked on new studies of the scope to enhance human perception and action in space using new sensory signals. In an initial study (Negen et al. 2018b), in a VR environment, we trained healthy adults to use an echo-like auditory cue, together with a noisy visual cue, to judge distance to an object. Within five short (approx. 1-h) training sessions, we found evidence for efficient Bayes-like combination, including improved precision (albeit falling short of the Bayes-optimal improvement) and reweighting with changing cue reliabilities. Recalling that children often do not show combination even with familiar, natural cues (Nardini et al. 2008b), this suggests that the mature perceptual-cognitive system may bring some advantages to novel cue combination problems and offers a promising outlook on flexibly enhancing human spatial abilities. However, many questions remain—including the prospects and training time course for eventually embedding such new abilities in low-level sensory processing, most likely to support subjectively effortless or ‘automatic’ perception.

### Enhancing human perception and action in space: future directions

Ongoing work is investigating the manner in which newly acquired spatial skills become embedded in perception. For example, there is initial evidence that within ten training sessions, and with another visual cue with a more natural form of noise (uncertainty), participants still do not attain Bayes-optimal performance; however, the skill enhances speed (as well as accuracy) of responses and resists verbal interference (Negen et al. 2021). Sensitive model-based tests of some of these abilities are assisted by analysis methods beyond those in the classic cue combination literature (Aston et al. 2021). Key future directions include investigating *extended training*, *neural substrates* (using fMRI), *motor/action tasks*, and *other perceptual problem domains* (e.g. sensing object properties, as well as their spatial locations).

## Summary and conclusions

The research described here has addressed two combination problems underlying perception in action in space: coordinating multiple reference frames and coordinating multiple sensory signals. Our understanding of development in these domains has been improved by adoption of a model-based approach, which, for example, compares performance with the predictions for an ideal (Bayesian) decision-maker. Both systems show substantial and extended development during childhood. In the domain of *reference frames*, key outstanding questions include the extent to which developmental improvements in abilities to either represent or select relevant information play a crucial role, and the extent to which these can be linked to maturation of specific brain systems and/or development of broader cognitive abilities. In the domain of *multiple sensory signals*, key outstanding questions include factors limiting efficient combination of signals in childhood, and the extent to which these can be tied to specific elements of information processing models and/or maturation of specific neural substrates. There are important parallels between the information processing challenges for *children using their familiar senses* and those for *adults learning to use new sensory signals*. Therefore, developmental research also has an important role in guiding the search for optimal approaches to enhancing human spatial abilities using technology.
